# GnRH dysregulation in polycystic ovarian syndrome (PCOS) is a manifestation of an altered neurotransmitter profile

**DOI:** 10.1186/s12958-018-0354-x

**Published:** 2018-04-11

**Authors:** Nirja Chaudhari, Mitali Dawalbhakta, Laxmipriya Nampoothiri

**Affiliations:** Reproductive-Neuro-Endocrinology Lab, Department of Biochemistry, Faculty of Science, The Maharaja Sayajirao University of Baroda, Vadodara, Gujarat India

**Keywords:** PCOS, GnRH, LH, FSH, Neurotransmitters

## Abstract

**Background:**

GnRH is the master molecule of reproduction that is influenced by several intrinsic and extrinsic factors such as neurotransmitters and neuropeptides. Any alteration in these regulatory loops may result in reproductive-endocrine dysfunction such as the polycystic ovarian syndrome (PCOS). Although low dopaminergic tone has been associated with PCOS, the role of neurotransmitters in PCOS remains unknown. The present study was therefore aimed at understanding the status of GnRH regulatory neurotransmitters to decipher the neuroendocrine pathology in PCOS.

**Methods:**

PCOS was induced in rats by oral administration of letrozole (aromatase inhibitor). Following PCOS validation, animals were assessed for gonadotropin levels and their mRNA expression. Neurotrasnmitter status was evaluated by estimating their levels, their metabolism and their receptor expression in hypothalamus, pituitary, hippocampus and frontal cortex of PCOS rat model.

**Results:**

We demonstrate that GnRH and LH inhibitory neurotransmitters – serotonin, dopamine, GABA and acetylcholine – are reduced while glutamate, a major stimulator of GnRH and LH release, is increased in the PCOS condition. Concomitant changes were observed for neurotransmitter metabolising enzymes and their receptors as well.

**Conclusion:**

Our results reveal that increased GnRH and LH pulsatility in PCOS condition likely result from the cumulative effect of altered GnRH stimulatory and inhibitory neurotransmitters in hypothalamic-pituitary centre. This, we hypothesise, is responsible for the depression and anxiety-like mood disorders commonly seen in PCOS women.

## Background

The reproductive system is governed by the hypothalamic-pituitary-gonadal axis (HPG), wherein a pulsatile release of GnRH from the hypothalamus stimulates anterior pituitary gonadotropes to release LH and FSH, leading to steroid production from the ovaries. The regulation of HPG axis is quite complex, involving several intrinsic factors (estrogens, progesterone, inhibin, activin, etc) [1] as well as extrinsic factors (neurotransmitters, neuropeptides, stress, etc) [2]. However, any abnormality that prevents or interferes with the function of these factors may culminate into reproductive endocrine anomalies. One of the most prevalent reproductive endocrinopathies is polycystic ovarian syndrome (PCOS), affecting 6–10% of women worldwide [3]. The key features of PCOS include hyperandrogenemia, oligo−/anovulation and peripheral cyst formation in ovaries [4]. In addition, PCOS is a disorder underpinning neuroendocrine abnormalities, characterized by increased GnRH and LH:FSH ratio [5]. However, in spite of its widespread occurrence and profound implications, the etiology of this disease remains poorly understood.

While reduced norepinephrine, dopamine and serotonin has been reported in the serum of PCOS women [6], their levels in GnRH regulatory regions of the brain are unknown due to the obvious difficulties in obtaining these tissues from patients. A tissue-specific understanding of the neurotransmitters would help us gain an insight into the pathogenesis of PCOS. Thereby, the objective of the current study was to evaluate the status of GnRH-regulatory neurotransmitters in a PCOS rat model.

To address the above objective, Letrozole, an aromatase inhibitor, was used to induce PCOS in rats [7]. Evaluation of the neurotransmitter levels was performed from hypothalamus, pituitary as well as from hippocampus and frontal cortex. The reasons for selecting these areas of brain mainly include i) presence of GnRHR in the described tissues, which contributes to regulation of reproduction and reproductive behaviour [8, 9]; ii) active steroidogenesis occurring in these regions [10, 11] and iii) them being important sites of neurotransmitter synthesis [12]. The rates of neurotransmitter synthesis and clearance were monitored by estimating the activities of neurotransmitter metabolizing enzymes. Gene expression analysis of specific neurotransmitter receptors that profoundly influence pulsatile release of GnRH/LH and other reproductive processes was performed in PCOS and normal rats.

## Methods

### Animals

Charles Foster female rats (2–3 months old) were housed in controlled conditions of temperature, humidity and light with ad libitum availability of food and water. Animals were allowed to acclimatize for one week before treatment. All experimental protocols listed herein were approved by the Institutional animal ethical committee (IAEC), Department of Biochemistry, The M. S. University of Baroda, India and they are in accordance with the ethical standards of the Committee For the Purpose of Control and Supervision of Experiments on Animals (CPCSEA), India.

### Induction of PCOS in rats

PCOS was induced in rats by oral administration of letrozole, a non-steroidal aromatase inhibitor [7]. For PCOS induction, 100 rats were randomly assigned to two different groups (*n* = 50 per group). A daily treatment regime of 21 days included oral administration through gavage of 0.5 ml of 1% carboxymethyl cellulose (CMC) for control group and 0.5 mg/kg body weight of letrozole dissolved in CMC for PCOS group. After 21 days of treatment, body weight, oral glucose tolerance, estrus cyclicity, serum estrogen, progesterone and testosterone levels and ovarian histology profile were analysed to check for development of PCOS.

### Estrus cyclicity

Estrus cyclicity was monitored daily before (for 14 days) and also during (for 21 days) the course of treatment by microscopic examination of the predominant cell type in vaginal smears [13]. Animals showing regular cycles of 4–5 days complete with the proestrus, estrus, metestrus and diestrus stages were defined as normal cyclic rats, whereas animals in which the estrus cycle was found arrested in any one of the stages for 4 consecutive days were termed as acyclic rats.

### Oral glucose tolerance test (OGTT)

OGTT was performed according to the method of Buchanan et al. [14], wherein 12 h fasting blood plasma was collected from orbital sinus into vials containing sodium fluoride and EDTA, followed by oral administration of glucose at 1 g/kg body weight. The blood was then collected every 30 min for 2 h and plasma glucose levels were estimated using Glucose oxidase-peroxidase (GOD-POD) kit (Reckon diagnostics, Vadodara, India).

### Blood and tissue collection

Blood and tissue collection was performed during diestrus stage of estrus cycle and between 8 and 9 AM in the morning. Blood was withdrawn through orbital sinus in a tube and centrifuged at 5000 g for 15 min at room temperature. Supernatant containing serum was separated and immediately stored at − 80 °C. Following blood collection, animals were euthanized by cervical dislocation. Pituitary, hypothalamus, hippocampus and frontal cortex were dissected out and stored at − 80 °C.

### Hormone profile

For estimation of hormones, commercially available ELISA kits were used (17β Estradiol ELISA kit-DKO003; Testosterone ELISA kit-DKO002 and Insulin ELISA kit-DKO076; Diametra. Italy). Progesterone was measured through ELISA kit (CAN-P-35) from Diagnostics Biochem Canada Inc., Canada. FSH and LH were estimated in serum using ELISA kits (rat FSH ELISA kit-E-EL-R0391; rat LH ELISA kit-E-EL-R0026) from Elabscience Biotechnology Co., Ltd., USA. Assays were performed according to manufacturers’ protocols. Sensitivity of methods are 8.68 pg/ml (17β Estradiol), 0.01 ng/ml (Testosterone), 0.1 ng/ml (Progesterone), 0.025 μIU/ml (Insulin), 0.38 ng/ml (FSH) and 0.19 ng/ml (LH) at 95% confidence limit.

### Histology

Ovaries of 6 different animals from each group were removed and fixed in Bouin’s fixative. For histological examination, 5 μm thick sections were prepared and stained with Hematoxylin-Eosin and histo-anatomical changes were observed under light microscope [15].

### Neurotransmitter estimation

Neurotransmitters were estimated using reverse phase HPLC coupled with electrochemical detector (Waters 2465; Waters corporation, Milford, USA) [16]. Tissues were homogenized in 0.17 M perchloric acid, centrifuged at 12000 g for 20 min at 4 °C and supernatant was immediately used for neurotransmitter estimation or kept at − 80 °C until use. For HPLC analysis, tissue samples as well as neurotransmitter standards were mixed with derivatization reagent (37 mM orthopthalaldehyde, 50 mM sodium sulfite, 90 mM tetraborate buffer-pH 10.4 and 5% methanol) for 10 min and 20 μl of sample was injected in HPLC. Glutamate and GABA were separated using a Sunfire® C18 column containing 0.1 M monosodium phosphate, 0.5 mM EDTA and 25% (*v*/v) methanol (pH 4.5) as mobile phase. For separation of norepinephrine, dopamine and serotonin, mobile phase containing a solution (pH 4.2) of 32 mM citric acid, 12.5 mM disodium hydrogen orthophosphate, 0.5 mM octyl sodium sulfate, 0.5 mM EDTA, 2 mM KCl and 15% (v/v) methanol was used. Standard curves were used to quantify the amount of neurotransmitter in each sample by calculating area under the curve (AUC).

### Epinephrine estimation

For epinephrine estimation, a colorimetric method was used [17]. Tissues were homogenized in 10% trichloroacetic acid followed by centrifugation at 10000 g for 10 min at 4 °C. Supernatant (0.2 ml) was added to a tube containing 0.25 ml of 10% (*w*/*v*) sodium carbonate and incubated for 30 min at room temperature followed by addition of 0.125 ml of Folin’s reagent and 0.375 ml of 5% (w/v) NaOH. Absorbance of epinephrine was recorded at 486 nm within 90 s of incubation.

### 5-Hydroxy tryptophan decarboxylase (TDC)

TDC was measured spectroflourimetrically as described by Sangwan and group [18]. Tissue homogenates were prepared in 0.1 M sodium phosphate buffer (pH 7.5) containing 5 mM thiourea, 1 mM EDTA and 5 mM β-mercaptoethanol. Tubes containing homogenates were centrifuged at 10000 g for 10 min at 4 °C, supernatant was separated and used as an enzyme source. For TDC assay, 0.1 ml of homogenate was added to a tube containing 0.7 ml of assay buffer (0.1 M sodium phosphate buffer-pH 8.5, 10% glycerol and 5 mM β-mercaptoethanol), 0.1 ml of 10 mM 5-hydroxytryptophan and 0.1 ml of 10 mM pyridoxal phosphate (PLP). The solutions were mixed properly and incubated at 37 °C for 40 min, followed by termination of enzyme reaction by adding 2 ml of 4 N NaOH. Serotonin formed was extracted by adding 3.5 ml of ethyl acetate followed by centrifugation at 1000 g for 10 min. Fluorescence measurement of upper organic layer was taken at 350 nm with prior excitation at 280 nm using a Hitachi F-7000 fluorescence spectrophotometer.

### GABA-transaminase (GABAT)

GABAT was estimated by kinetic method using spectrophotometer [19]. Tissues were homogenized in phosphate buffer (50 mM NaH_2_PO_4_, 5 mM KCl, 120 mM NaCl, pH 7.4) and centrifuged at 12,000 g for 15 min at 4 °C. The pellet was resuspended in phosphate buffer and was used for enzyme assay. Tissue homogenates (50 μl) were incubated with 1 ml of 100 mM potassium pyrophosphate buffer (pH 8.6) containing 5 mM α-ketoglutarate, 4 mM NAD, 3.5 mM β-mercaptoethanol and 10 μM pyridoxal phosphate for 15 min at 37 °C. The absorbance of blank was measured at 340 nm followed by addition of 0.1 ml of 100 mM GABA. The absorbance was immediately monitored at 340 nm for every 10 s for 2 min.

### Glutamic acid decarboxylase (GAD)

GABA formed by the enzyme GAD was estimated using spectroflourimetry method [20]. Tissues were homogenized in 0.15 M KCl containing 5 mM EDTA and 0.5% triton-X, incubated for 30 min on ice and centrifuged at 3000 g for 10 min. Supernatant (0.1 ml) was incubated with a solution containing 80 mM potassium phosphate buffer (pH 6.2), 25 mM sodium glutamate and 0.5 mM pyridoxal phosphate for 30 min at 37 °C.The reaction of GAD was terminated by addition of 0.5 ml of 15% TCA followed by centrifugation at 5000 g for 10 min. GABA containing supernatant was mixed with 0.5 ml of 14 mM ninhydrin solution and the tubes were kept in a water-bath set at 60 °C for 30 min. The samples were incubated with 5 ml of copper tartarate reagent (160 mg sodium bicarbonate, 30 mg copper sulphate and 33 mg tartaric acid dissolved in 100 ml of distilled water) for 15 min. The fluorescence emission was measured at 451 nm with prior excitation at 377 nm using a Hitachi F-7000 fluorescence spectrophotometer.

### Monoamine oxidase (MAOA & MAOB)

Spectrophotometric method was employed for estimation of MAOA and MAOB activity [21]. Tissue homogenates were prepared in homogenate buffer (0.25 M sucrose, 20 mM EDTA and 0.1 M tris- pH 7.4) and cell debris was removed by centrifugation at 800 g for 10 min at 4 °C. The supernatant was centrifuged at 12000 g for 20 min at 4 °C and resultant pellet was dissolved in 0.01 M sodium phosphate buffer (pH 7.4) containing 320 mM sucrose by keeping in ice for 20 min. The tubes were again centrifuged at 3000 g for 10 min at 4 °C and supernatant obtained was used as enzyme source. The assay mixture for MAOA included 0.1 M sodium phosphate buffer (pH 7.4) containing 0.4 mM 5-hydroxytryptamine whereas for MAOB 10 mM of benzylamine was used as a substrate. The reaction was terminated by addition of 0.2 ml of 1 M HCl after 20 min of incubation at 37 °C. Product formed was extracted by vortexing for 5 min with 2 ml of butyl acetate for MAOA or cyclohexane for MAOB. Tubes were centrifuged at 3000g for 5 min and upper organic layer was measured at wavelength of 280 nm for MAO-A activity and 242 nm for MAO-B activity with spectrophotometer, respectively.

### Glutamate dehydrogenase (GDH)

GDH activity was measured in the direction of oxidative deamination of glutamate into α-ketoglutarate [22]. Tissues were homogenized in 10 volume of 0.25 M sucrose-10 mM HEPES, pH -7.4 and centrifuged at 1000 g for 10 min at 4 °C. The supernatant was collected in a fresh vial and centrifuged at 12000 g for 30 min at 4 °C to yield mitochondrial pellet which was dissolved in homogenate buffer and used as enzyme source. The enzyme (0.05 ml) was added to assay mixture (50 mM tris buffer-pH 9.5, 1 M glutamate, 0.1 M EDTA, 56 mM NAD and 40 mM ADP) and absorbance was monitored immediately at 340 nm for every 10 s for 1 min.

### Acetylcholine esterase (AChE)

Acetylcholine is degraded by enzyme AChE, which was estimated by kinetic method [23]. Tissue homogenates were prepared in 0.1 M sodium phosphate buffer (pH 8.0), centrifuged at 12000 g for 5 min at 4 °C and resulting supernatant was used as enzyme source. Tubes containing 1.5 ml of 0.1 M phosphate buffer (pH 8.0), 0.01 ml of freshly prepared substrate (75 mM acetylcholine iodide in distilled water) and 0.05 ml of freshly made Ellman’s reagent (10 mM DTNB and 17.85 mM NaHCO_3_ dissolved in 0.1 M sodium phosphate buffer – pH 7.0) were incubated at 25 °C. Absorbance of blank was measured at 405 nm followed by addition of 0.2 ml of enzyme. The change in absorbance was monitored thereafter for 10 min at every 2 min-interval.

### RNA isolation and PCR

Total RNA was extracted using TRIsoln reagent (GeNei, India) and 2 μg of RNA was reverse-transcribed using Verso cDNA synthesis kit with Oligo-dT primers (ThermoScientific, USA). Real-time quantitative polymerase chain reaction (qPCR) was performed using Quantstudio Real Time PCR system (Life Technologies, USA). Primers were procured from IDT (CA, USA) and their sequences are given in Table 1. All the samples were run in triplicate and accompanied by a non-template control. PCR was performed with SYBR select PCR Master Mix (Applied Biosystems, USA) according to manufacturer’s protocol. Thermal cycling conditions included initial denaturation in one cycle of 15 min at 95 °C, followed by 45 cycles of 15 s at 94 °C, 30 s at 60 °C and 30 s at 72 °C. The fold changes in the mRNA were calculated for each sample group using the 2^-ΔΔCT^ method [24]. Fold changes in expression of less than 0.5 and greater than 2 were considered biologically significant.

**Table 1:** Primer sequences of rat targeted genes

Targeted Genes	Primer sequence	Accession number
*GnRH1*	F: 5’ CCGCTGTTGTTCTGTTGACTG 3′ R: 5’ TCACACTCGGATGTTGTGGA 3′	NM_012767
*GnRHR*	F: 5’ TCTGCAATGCCAAAATCATC 3′ R: 5’ GTAGGGAGTCCAGCAGATGAC 3′	NM_031038.3
*FSHβ*	F: 5’ AGGAAGAGTGCCGTTTCTGC 3′ R: 5’ GCTGTCACTATCACACTTGC 3′	NM_001007597.2
*LHβ*	F: 5’ CTGTCCTAGCATGGTTCGAGT 3′ R: 5’ AGTTAGTGGGTGGGCATCAG 3′	NM_012858.2
*TH*	F: 5’ CATTGGACTTGCATCTCTGG 3′ R: 5’ GTTCCTGAGCTTGTCCTTGG 3′	NM_012740.3
*COMT*	F: 5’ GACGCGAAAGGCCAAATCAT 3′ R: 5’ ACGTTGTCAGCTAGGAGCA 3′	NM_012531.2
*5HT1A*	F:5’CCCCCCAAGAAGAGCCTGAA3′ R:5’GGCAGCCAGCAGAGGATGAA3′	NM_012585.1
*α1AR*	F: 5’ ACCAGCTCCGGTGAACATTT 3′ R: 5’ GCCGCCCAGATATTGCAGAA 3′	NM_017191.2
*D2R*	F:5’ TGAACCTGTGTGCCATCAGCA 3′ R:5’ TTGGCTCTGAAAGCTCGACTG 3′	NM_012547.1
*GABAB1*	F:5’CGCTACCATCCAACAGACCA3′ R:5’TGTCAGCATACCACCCGATG3′	NM_031028.3
*M2-AchR*	F:5’CACAGTTTCCACTTCGCTGG 3′ R:5’ CACCTTTTTGGGCCTTGGTG 3′	NM_031016.1
*NMDAR*	F:5’ ACACCGACCAAGAAGCCATC 3′ R:5’ GGACTCATCCTTATCCGCCA 3	NM_012574.1
*β-Actin*	F: 5’ AGGCCCCTCTGAACCCTAAG 3′ R: 5’ GGAGCGCGTAACCCTCATAG 3′	NM_031144.3

### Statistical analysis

Statistical analysis was performed using Student’s t-test and Two-way ANOVA, followed by Bonferroni *post-hoc* test using GraphPad Prism 5 software. *P* values of < 0.05 were deemed to be statistically significant.

## Results

### Induction of PCOS in rats

**Fig. 1:**
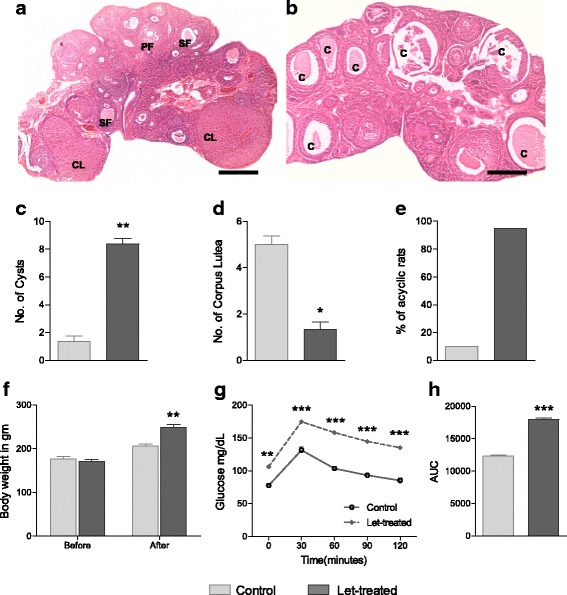
Validation of PCOS rat model after 21 days of letrozole treatment. Hematoxylin-Eosin stained ovarian sections of Control group (**a**) and letrozole treatment group (**b**) under 4X magnification (Scale bar = 500μm). PF: Primary follicle; SF: secondary follicle; CL: Corpus luteum; C: Cyst. **c** Number of cystic follicles, **d** number of CL, and **e** percent of females that were acyclic after letrozole treatment. Change in body weight (**f**); Oral glucose tolerance test profile (**g**) and area under the curve (AUC) for glucose tolerance (**h**) in control and treated rats. Error bars represent SEM; *n* = 6–10 per group; **P < 0.01; ***P < 0.001 as compared to control group

Testosterone levels were significantly elevated in serum of letrozole treated animals (*P* < 0.001) with a decrease in progesterone levels (*P* < 0.05) and no change in serum estradiol levels (Table 2). Figure 1 demonstrates the ovarian histology. Control sections showed follicles in various stages of development (Fig. 1a) whereas treatment group sections had numerous peripheral cysts along with low number of corpus lutea (Fig. 1b, c and d). Treatment group also had a high percentage of acyclic rats (disturbed estrus cycle) (Fig. 1e); mainly arrested in diestrus stage. Further, there was a significant increase in the body weight (Fig. 1f) (*P* < 0.01), glucose intolerance (Fig. 1g), area under the curve for glucose (Fig. 1h), serum insulin level (*P* < .001) and HOMA-IR index (Table 2) of letrozole treated group as compared to the CMC control group. All the animals of letrozole-treated group exhibited hormonal and structural alterations and were considered as PCOS positive rats for further experiments.

**Table 2:** Serum hormone profile after 21 days of letrozole treatment

	Estradiol (pg/ml)	Testosterone (ng/ml)	Progesterone (ng/ml)	Insulin(μIU/ml)	HOMA-IR
Control	132.3 ± 17.37	0.315 ± 0.05	12.25 ± 0.54	7.42 ± 1.04	1.56 ± 0.14
Let-treated	139.3 ± 19.10	1.177 ± 0.07***	9.95 ± 0.39*	15.54 ± 1.27***	4.40 ± 0.21***

All values are presented as Mean ± SEM; *n* = 6 per group; *P < 0.05; ***P < 0.001 as compared to control

$$ \mathrm{HOMA}\kern0.5em \hbox{-} \kern0.5em \mathrm{IR}\kern0.5em =\kern0.5em \frac{\left[\mathrm{Glucose}\kern0.5em \left(\mathrm{mg}/\mathrm{dl}\right)\kern0.5em \times \kern0.5em \mathrm{Insulin}\kern0.5em \left(\upmu \mathrm{IU}/\mathrm{ml}\right)\right]\kern0.5em }{405} $$

### Gonadotropin status in PCOS

**Fig. 2:**
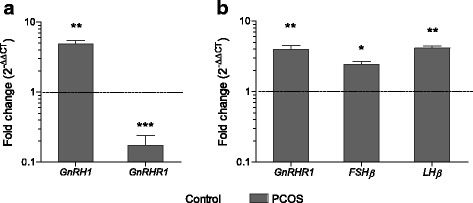
Gonadotropin status in letrozole-induced PCOS animals. **a** Expression profile of hypothalamic *GnRH1, GnRHR1*, and **b** pituitary *GnRHR1, FSHβ* and *LHβ*. *β-Actin* was used as internal control and fold change in expression was calculated by 2^-ΔΔCT^ method. Values are mean fold change in gene expression of PCOS group samples as compared to control samples (represented by black dashed line). Error bars represent SEM; *n* = 6 per group; **P < 0.01; ***P < 0.001 as compared to control group

*GnRH1* plays a pivotal role in stimulating pituitary release of FSH and LH. When analysed for gene expression (Fig. 2a), PCOS rats demonstrated significantly increased transcripts of hypothalamic *GnRH1* (*P* < 0.01) and pituitary *GnRHR* (P < 0.01), while hypothalamic *GnRHR* expression was reduced (*P* < 0.001) as compared to control rats. GnRH released from the hypothalamus stimulates gonadotropin secretion from anterior pituitary. Gonadotropin estimations revealed no change in FSH levels among both the groups while a significant increase was observed in LH levels of PCOS rats (*P* < 0.001), leading to an elevated LH:FSH ratio (Table 3). To examine whether the origin of this alteration lies at the genetic level, transcript analysis was carried out. In present study, both the *FSHβ* (*P* < 0.05) as well as *LHβ* (*P* < 0.01) mRNA were found significantly increased in the pituitary of PCOS rats as compared to control (Fig. 2b).

**Table 3:** Serum Gonadotropin levels in letrozole induced PCOS rat model

	FSH (ng/ml)	LH (ng/ml)	LH: FSH
Control	2.20 ± 0.099	2.40 ± 0.138	1.08: 1
PCOS	2.29 ± 0.180	6.76 ± 0.132 ***	2.97: 1***

All values are presented as Mean ± SEM; *n* = 6 per group; ***P < 0.001 as compared to control values

### Neurotransmitter levels in PCOS rats

**Fig. 3:**
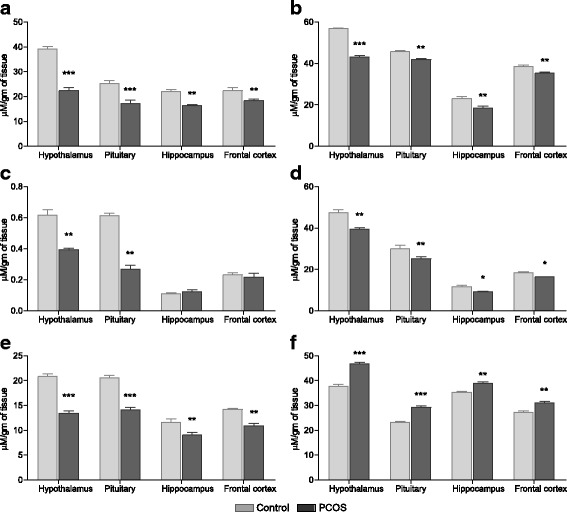
Neurotransmitter levels in control and PCOS animals. **a** Serotonin, **b** norepinephrine, **c** epinephrine, **d** dopamine, **e** GABA and **f** glutamate levels in different tissues of control and PCOS rats. All values are represented as Mean ± SEM; *n* = 6 per group; *P < 0.05; **P < 0.01; ***P < 0.001 as compared to control group

GnRH and LH release are mainly influenced by various neurotransmitters secreted by discrete brain regions. When estimated, serotonin levels (Fig. 3a) were significantly reduced in all the tissues analysed with greatest decrease in hypothalamus (*P* < 0.001) and pituitary (*P* < 0.001) of PCOS group rats as compared to control. A similar trend was observed for norepinephrine (Fig. 3b) content. Epinephrine levels (Fig. 3c) were also decreased in hypothalamus (*P* < 0.01) and pituitary (P < 0.01) of PCOS animals; however no difference was observed in hippocampus and frontal cortex. Furthermore, notably low levels of dopamine (Fig. 3d) and GABA (Fig. 3e) were also seen in all tested brain tissues of PCOS animals as compared to control rats. In contrast to all these neurotransmitters, the amount of glutamate was significantly elevated (Fig. 3f) in hypothalamus (*P* < 0.001), pituitary (*P* < 0.001), hippocampus (*P* < 0.01) and frontal cortex (*P* < 0.01) of PCOS rats as compared to control.

### Neurotransmitter synthesizing enzymes in PCOS rats

**Fig. 4:**
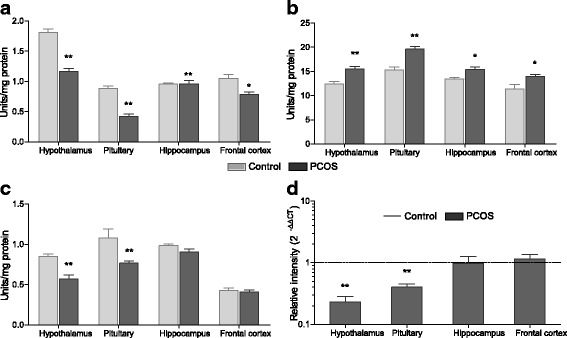
Status of neurotransmitter synthesizing enzymes. Activity of **a** tryptophan decarboxylase (TDC), **b** GABA transaminase (GABA-T), and **c** glutamic acid decarboxylase (GAD) in different tissues of control and PCOS rats. **d** Values are mean fold change in tyrosine hydroxylase (*TH*) mRNA expression of PCOS group samples as compared to control samples (represented by black dashed line). *β-Actin* was used as internal control for mRNA studies and fold change in expression was calculated by 2^-ΔΔCT^ method. Error bars represent SEM; *n* = 6 per group; *P < 0.05; **P < 0.01; ***P < 0.001 as compared to control group

The turnover of neurotransmitters is tightly regulated by the activities of their metabolising enzymes. Serotonin synthesizing enzyme Tryptophan decarboxylase (TDC) was reduced in all analysed brain tissues of PCOS animals (*P* < 0.01), except in the hippocampus (Fig. 4a). GABA-T, which acts as a synthesizing enzyme for glutamate and degrading enzyme for GABA, showed heightened activity in hypothalamus (P < 0.01), pituitary (P < 0.01), hippocampus (*P* < 0.05) and frontal cortex (P < 0.05) of PCOS rats as compared to control animals (Fig. 4b). Glutamic acid decarboxylase (GAD) enzyme catalyses the conversion of glutamate into GABA. GAD activity exhibited notable decrease in hypothalamus (*P* < 0.01) and pituitary (P < 0.01) of PCOS rats but no change was observed in other tissues (Fig. 4c). Furthermore, gene expression of tyrosine hydroxylase *(TH)*, a rate limiting enzyme for all catecholamine synthesis, was reduced in hypothalamus (*P* < 0.01) and pituitary (*P* < 0.05) of PCOS rats as compared to control (Fig. 4d).

### Neurotransmitter degrading enzymes in PCOS rats

**Fig. 5:**
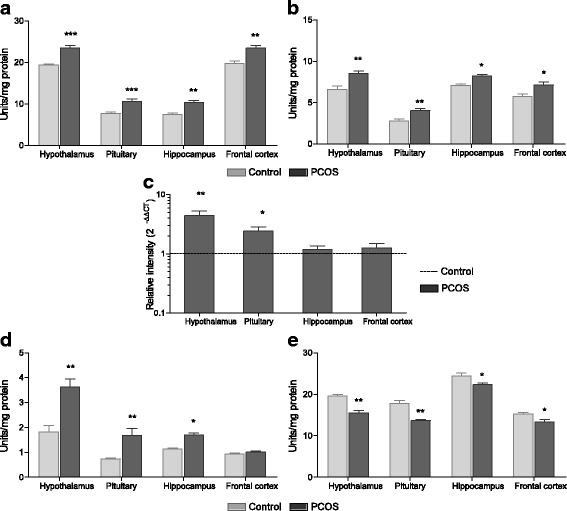
Status of neurotransmitter degrading enzymes in PCOS rats. Enzyme activity of **a** monoamine oxidase A (MAOA) and **b** MAOB in various tissues of control and PCOS rats. **c** Bar graph represents values of mean fold change in gene expression of Catechol-*O*-methyl transferase (*COMT*) in PCOS animals as compared to control rats (represented by black dashed line). *β-Actin* was used as internal control for mRNA studies and fold change in expression was calculated by 2^-ΔΔCT^ method. Activity of **d** acetylcholine esterase (AChE) and **e** glutamate dehydrogenase (GDH) in tissues of control and PCOS animals. Error bars represent SEM; *n* = 6 per group; *P < 0.05; **P < 0.01; ***P < 0.001 as compared to control group

Neurotransmitters are quickly metabolized through degrading enzymes secreted into the synaptic cleft. Serotonin, norepinephrine, dopamine and epinephrine are metabolized by monoamine oxidase (MAO). When analysed for activity, a significant increase in MAO-A was observed in hypothalamus (*P* < 0.001), pituitary (*P* < 0.001), hippocampus (*P* < 0.01) and frontal cortex (*P* < 0.01) of PCOS rats as compared to control animals (Fig. 5a). MAO-B also followed a similar trend in all the tissues but it was less obvious as compared to MAO-A activity (Fig. 5b). Another major enzyme which degrades catecholamines dopamine, norepinephrine and epinephrine is Catechol-*O*-methyl transferase (COMT). The transcript level of *COMT* was markedly elevated in hypothalamus (*P* < 0.01) and pituitary (*P* < 0.05) of PCOS rats while no change was observed in other tissues (Fig. 5c). In addition, metabolizing enzymes glutamate dehydrogenase (GDH) and acetylcholine esterase (AChE), which degrade glutamate and acetylcholine respectively, were also assessed. Acetylcholine esterase activity (Fig. 5d) was also higher in PCOS animals as evident in hypothalamus (*P* < 0.01), pituitary (*P* < 0.01) and hippocampus (*P* < 0.05). In contrast to other metabolizing enzymes activity, that is increased in PCOS animals, GDH activity (Fig. 5e) was significantly low in hypothalamus (*P* < 0.01), pituitary (*P* < 0.01), hippocampus (*P* < 0.05) and frontal cortex as compared to control tissues.

### Neurotransmitter receptor profile in PCOS rats

**Fig. 6:**
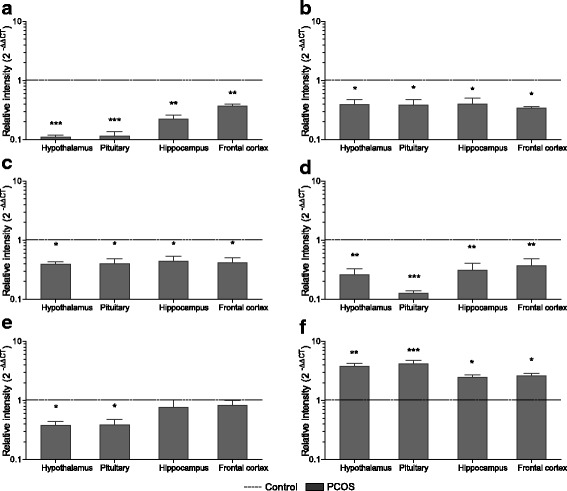
Transcript analysis of neurotransmitter receptor in PCOS rats. mRNA expression profile of **a** serotonin 5HT1A receptor (5HT_1A_); **b** alpha1-adrenergic receptor (*α*_*1*_*AR*); **c** dopamine D_2_ receptor (*D*_*2*_*R*); **d** GABA B1 receptor *(GABA*_*B1*_*)*; **e** muscarinic acetylcholine 2 receptor *(M*_*2*_*AchR)* and **f** NMDA glutamate receptor *(NMDA)* in control and PCOS brain tissues. Bar graph represents values of mean fold change in gene expression of PCOS animals as compared to control rats (represented by black dashed line). *β-Actin* was used as internal control for mRNA studies and fold change in expression was calculated by 2^-ΔΔCT^ method. Error bars represent SEM; *n* = 6 per group; *P < 0.05; **P < 0.01; ***P < 0.001 as compared to control group

Neurotransmitter receptors expressed on pre- or postsynaptic neurons relay their biological action. An mRNA expression profile of various neurotransmitter receptors was thus generated using quantitative real-time PCR. Transcript levels for serotonin receptor *5HT1A*, adrenergic alpha1 receptor *(α1AR)*, dopamine D2 receptor (*D2R)* and *GABAB1* receptor declined significantly in all tissues tested in PCOS rats as compared to control (Fig. 6a-d). Transcriptional down-regulation of muscarinic acetylcholine 2 receptor (*M2AchR)* receptor (Fig. 6e) was also observed in hypothalamus (P < 0.05) and pituitary (*P* < 0.05) of PCOS animals. Contrary to all these and in line with glutamate content, *NMDA* receptor expression (Fig. 6f) was found markedly high in hypothalamus (*P* < 0.01), pituitary (*P* < 0.001), hippocampus (*P* < 0.05) and frontal cortex (*P* < 0.05) of PCOS animals when compared with control tissues.

## Discussion

PCOS is a very common endocrine disorder in women of reproductive age and is characterized by increased androgen production and abnormal gonadotropin secretion, resulting in chronic anovulation. To understand the etiopathology of PCOS, the present study employed a letrozole-treated rat model, which exhibits hormonal, reproductive and metabolic signs similar to the human PCOS condition [7, 25, 26]. Furthermore, it was observed currently that the PCOS rats had a high serum LH:FSH ratio, a characteristic feature of PCOS. This, we believe, must stem from increased transcription of the *GnRH* in the hypothalamus and *GnRHR* in the pituitary, as also observed by Kauffman and group [26]. Thus, letrozole-induced PCOS rat model possesses similar neuroendocrine traits as seen in PCOS women, making it a favourable model for use in PCOS research.

A number of studies using dual-label immunohistochemistry and in situ hybridization have shown that several neurotransmitter and neuropeptide receptors are expressed in GnRH neurons and they directly regulate GnRH, LH and FSH release [2]. The effect of serotonin on GnRH neurons is biphasic in nature wherein activation of 5-HT2A receptor increases GnRH neuronal activity via PKC (Protein kinase C) pathway, while activation of 5-HT1A receptor suppresses GnRH neuronal firing through adenylate cyclase [27, 28]. Serotonin content was found significantly reduced in all brain tissues of PCOS animals as compared to control, which can be well correlated with the observed decrease in TDC activity (serotonin synthesis) and heightened MAO activity. Also, the expression of 5HT1A receptor was decreased in PCOS animals. Based on this, and the above-cited references, increased GnRH and LH release in PCOS may result, at least partially, from reduced inhibition of GnRH by serotonin.

In addition to serotonin, the role of catecholamines is also known in GnRH regulation. Norepinephrine has been shown to rapidly increase GnRH mRNA levels in ovariectomised rats [29]. It is also responsible for the pre-ovulatory LH surge through the α- and β-adrenergic receptors. Propranolol, an α-adrenergic receptor blocker stimulates NE-induced LH release, while treatment of β-antagonist blocked the release of pre-ovulatory LH surge [30, 31]. This indicates that the stimulatory effect of norepinephrine on LH release is mediated by β-adrenergic receptors while α-adrenergic receptor inhibits LH release. Moreover, α-adrenergic receptor is also involved in steroid mediated feedback regulation of GnRH [32]. In the case of epinephrine, although reports on GnRH regulation are rare, a positive relation is implied, where the former stimulates release of GnRH and LH, also through the α-adrenergic receptor [33, 34]. Whereas both epinephrine and norepinephrine were reduced in the brain of PCOS rats, the GnRH and LH were still elevated, pointing towards the involvement of other regulatory factors in this outcome.

Unlike norepinephrine and epinephrine, dopamine is a major suppressor for GnRH release [35]. It also inhibited the firing and anteroventral paraventricular (AVPV)-evoked GABA/glutamate postsynaptic currents in the GnRH neurons in vitro mediated by D1 and D2-like receptors in male and female mice [35]. Recent study in ewes also suggests that D2 dopamine receptor not only affects the GnRH release but also GnRH and GnRHR gene expression in hypothalamus. Also, LH pulse frequency increases upon local injection of Sulpride (D2R antagonist) in ewes, reflecting the potency of D2R in inhibiting GnRH and LH pulsatility [36]. In addition to its influence on GnRH/LH, it has an inhibitory effect on prolactin release. A positive association between PCOS, low dopamine and hyperprolactinemia has been suggested [37]. Further, hyperprolactinemia exerts an inhibitory effect on the gonadotrophs [38]. In the present case, dopamine content in brain of PCOS rats was significantly decreased along with reduced expression of *D2R*, which may result into hypersecretion of prolactin in PCOS condition. Supporting our data, many studies suggest the role of reduced dopaminergic tone in increased LH release in PCOS [39, 40]. Additionally, treatment with bromocriptine, a D2 receptor agonist, can restore normal menstrual cycle and ovulation in PCOS women [39].

GABA is the major inhibitory neuron of the central nervous system. GABAB1 knockout mice demonstrated abnormal estrus cyclicity and reduced fecundity with significantly increased GnRH release as well as GnRH pulse frequency [41], whereas GABAA knockdown mice had normal estrus cycle and puberty onset [42]. In addition, treatment of GABA or muscimol, a GABAA/C receptor agonist, to cultured anterior pituitary cells results into increased secretion of LH through Ca2+ release [43]. However, when cultured pituitary cells were incubated with baclofen, a GABAB agonist, GnRH-induced LH release was inhibited while basal LH secretion did not change [43, 44]. This suggests that GABAA/C stimulate basal LH secretion whereas GABAB suppresses GnRH-induced LH release. Reduced signalling of GABA through GABAB1 observed in our system may have contributed to an increased GnRH/LH pulse. Interestingly, a study in prenatally androgenised mouse model of PCOS demonstrated increased GABA innervations to GnRH neurons [45]. This disparity in the result is likely due to the fact that Moore and group have used arcuate nucleus for their study while we have used whole hypothalamus, which includes many such nuclei. Also, this group has used prenatally androgenised mouse model of PCOS [45] and in utero androgen exposure can lead to epigenetic changes, which could result in developmental alterations in neural circuits.

In contrast to GABA, Glutamate is the major excitatory neurotransmitter for GnRH release. GnRH neurons express both ionotropic glutamate receptors (AMPA, Kainate and NMDA) and metabotropic glutamate receptors. However, reports describing the role of metabotropic glutamate receptors in GnRH regulation are scanty [46]. NMDA receptor antagonist-MK801 abolished endogenous pulses of GnRH secretion whereas pulsatile release of GnRH was not affected in the presence of 6,7-dinitroquinoxaline-2,3-dione (kainate receptor blocker) [47]. In addition, mRNA and protein expression study has revealed the presence of vesicular glutamate transporter in gonadotrophs of anterior pituitary and a stimulatory role for glutamate in LH release was also documented [48, 49]. In PNA-induced PCOS mouse model, no effect of glutamate was observed in GnRH pulsatility [45]. However, significantly high glutamate levels and NMDA receptor expression in PCOS animals were observed in the current study, suggesting direct overstimulation of GnRH and LH release. Further, the activities of GAD and GDH were significantly decreased in PCOS rats while that of GABA-T was markedly elevated suggesting that in PCOS condition the flux of reaction is towards the glutamate and not towards GABA.

Along with all the above-stated neurotransmitters, the role of acetylcholine in GnRH regulation is also emerging. In cultured GT1–7 cell line, acetylcholine stimulates GnRH release through activation of nicotinic receptor whilst an inhibitory effect of acetylcholine on GnRH activity was mediated by muscarinic receptor activation [50]. Also, in GT1–7 cells, acetylcholine treatment activates M2 muscarinic receptor that further reduces forskolin-induced cAMP production followed by suppression of GnRH release [51]. Similarly, treatment of exogenous acetylcholine to cultured anterior pituitary cells resulted in decreased response of GnRH-induced LH release. This response was counteracted by muscarinic receptor antagonist atropine [52]. Currently, activity of acetylcholine esterase (AChE), a hydrolytic enzyme of acetylcholine, was found elevated in the hypothalamus and pituitary of PCOS rats along with decreased expression of M2 muscarinic acetylcholine receptor (M2AChR), thus, suggesting reduced levels of acetylcholine in PCOS condition which may contribute to increased GnRH and LH pulse frequency in PCOS women.

It should be noted that along with neurotransmitters, HPG axis is governed by several neuropeptides of the like of kisspeptin, a major factor which directly or through its interaction with steroids and neurotransmitters, stimulates the release and expression of GnRH/LH [53, 54]. Various immnohistochemical studies have demonstrated co-localization of GABAB, NMDAR glutamate receptor and D2 dopamine receptor in kisspeptin neurons. Furthermore, antagonists of GABAB and D2R dopamine receptors increase Kisspeptin-mediated GnRH and LH release [55, 56], whereas treatment with MK801-NMDA receptor antagonist blocks kisspeptin-dependent reinstatement of LH surge [57]. Data from our lab has revealed significant increases in expression of both *Kiss1* and its receptor *Gpr-54* in the hypothalamus of PCOS rats (manuscript under preparation) that also falls in line with a previous study [26]. Thus, alteration of neurotransmitters and neuropeptide together are likely to be responsible for the increased GnRH and LH pulsatility in PCOS condition.

Besides the regulation of endocrine axis, neurotransmitters are also implicated in several psychiatric manifestations. The vast majority of anti-depressants include inhibitors of monoamine oxidase and serotonin reuptake transporters (SSRI), indicating the role of serotonin, dopamine and norepinephrine in mood regulation [58–61]. Glutamate and GABA are also emerging candidates for depression and anxiety disorders [62, 63]. Alterations in acetylcholine signalling have also been shown to lead to symptoms of depression and anxiety wherein overactive or hyper-responsive muscarinic cholinergic system has been documented [58]. All these references suggest that alteration in neurotransmitter profile seen in letrozole-induced PCOS model may result into development of depression and anxiety-like symptoms (manuscript communicated). In light of these references and our data, the occurrence of depression, anxiety and other mood disorders, which affect upto 40% of PCOS women [64, 65], can be linked to an altered neurotransmitter profile. We have indeed observed symptoms of depression and anxiety in behavioural experiments on letrozole-induced PCOS rat model (manuscript communicated).

Current study clearly demonstrated severe neurotransmitter modulation in letrozole-induced PCOS rat model. Likewise, in a study, rat treated with letrozole demonstrated decreased norepinephrine and dopamine content in hippocampus and frontal cortex [66]. However, the concentration and duration of letrozole treatment in that study was much higher as compared to our study. Furthermore, the possibility that the currently observed changes in neurotransmitters are due to other interactions of letrozole can be ruled out by our previous study wherein testosterone propionate-induced PCOS rat model also demonstrated similar neurotransmitter profile [67]. This thereby strengthens the result of present study indicating that neurotransmitter modulation is a pivotal attribute of PCOS condition.

## Conclusion

**Fig. 7:**
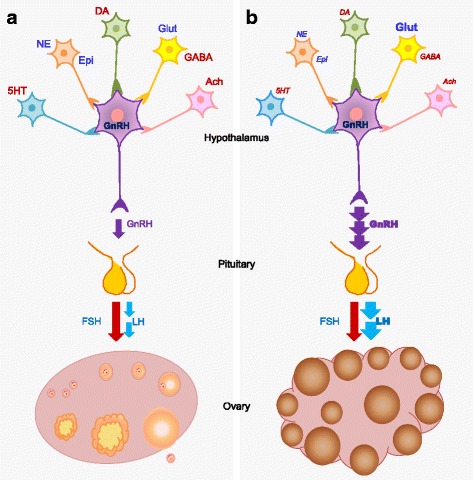
Diagrammatic summary of results. Hypothalamic-pituitary-ovarian axis in normal (**a**) and in PCOS (**b**) conditions. Blue font: Stimulatory molecules; Red font: Inhibitory molecules; Bold font: Increased in PCOS condition; *Italic font*: Decreased in PCOS condition. Thick arrow: increased in PCOS condition as compared to control. 5HT: Serotonin; DA: Dopamine; NE: Norepinephrine; Epi: Epinephrine; Glut: Glutamate; Ach: Acetylcholine

Results from our study thus suggest the presence of heightened excitatory signal (glutamate) and decreased inhibitory currents (serotonin, dopamine, GABA and acetylcholine), which may be responsible for the increased pulsatility of GnRH and LH, leading to increased LH/FSH ratio as observed in PCOS (Fig. 7). It is also evident that the observed changes in neurotransmitter levels of the brain are mainly due to altered rates of their catabolism. Further, the dysregulated neurotransmitter profile in PCOS could also be the reason for low self-esteem, anxiety, frequent mood swings and depression, features closely associated with PCOS women. This is the first study which explicitly demonstrates that neurotransmitter modulation may act as a key feature in the development of PCOS pathology with increasing risk of other co-morbidities such as stress and mood.

## Abbreviations

5HT_1A_: Serotonin 5HT_1A_ receptor
AChE: Acetyl cholinesterase
COMT: Catecholamine-O-methyl transferase
D_2_R: Dopamine D_2_ receptor
FSH: Follicle stimulating hormone
GABA: γ-amino butyric acid
GABA_B1_: GABA_B1_ receptor
GABA-T: GABA transaminase
GAD: Glutamic acid decarboxylase
GDH: Glutamate dehydrogenase
GnRH: Gonadotropin-releasing hormone
HOMA-IR: Homeostatic Model Assessment of Insulin Resistance
HPG axis: Hypothalamic-pituitary-gonadal axis
LH: Luteinizing hormone
M_2_AchR: Muscarinic acetylcholine 2 receptor
MAO: Monoamine oxidase
NMDA: N-methyl-D-aspartate glutamate receptor
OGTT: Oral glucose tolerance test
TDC: Tryptophan decarboxylase
TH: Tyrosine hydroxylase
α_1_AR: α_1_-adrenergic receptor

## References

[1] Levine JE. An introduction to neuroendocrine systems. Handbook of Neuroendocrinology. Academic press; 2011. p. 3.

[2] Smith MJ, Jennes L (2001). Neural signals that regulate GnRH neurones directly during the oestrous cycle. Reproduction.

[3] Joshi B, Mukherjee S, Patil A, Purandare A, Chauhan S, Vaidya R (2014). A cross-sectional study of polycystic ovarian syndrome among adolescent and young girls in Mumbai, India. Indian J Endocrinol Metab.

[4] Diamanti-Kandarakis E (2008). Polycystic ovarian syndrome: pathophysiology, molecular aspects and clinical implications. Expert Rev Mol Med.

[5] Azziz R, Carmina E, Chen Z, Dunaif A, Laven JS, Legro RS, Lizneva D, Natterson-Horowtiz B, Teede HJ, Yildiz BO (2016). Polycystic ovary syndrome. Nat Rev Dis Primers.

[6] Shi X, Zhang L, Fu S, Li N (2011). Co-involvement of psychological and neurological abnormalities in infertility with polycystic ovarian syndrome. Arch Gynecol Obstet.

[7] Kafali H, Iriadam M, Ozardalı I, Demir N (2004). Letrozole-induced polycystic ovaries in the rat: a new model for cystic ovarian disease. Arch Med Res.

[8] Badr M, Pelletier G (1987). Characterization and autoradiographic localization of LHRH receptors in the rat brain. Synapse.

[9] Maggi R (2016). Physiology of gonadotropin-releasing hormone (GnRH): beyond the control of reproductive functions. MOJ Anat Physiol.

[10] Furukawa A, Miyatake A, Ohnishi T, Ichikawa Y (1998). Steroidogenic acute regulatory protein (StAR) transcripts constitutively expressed in the adult rat central nervous system: Colocalization of StAR, cytochrome P-450SCC (CYP XIA1), and 3β-Hydroxysteroid dehydrogenase in the rat brain. J Neurochem.

[11] Zwain IH, Yen SS (1999). Neurosteroidogenesis in astrocytes, oligodendrocytes, and neurons of cerebral cortex of rat brain. Endocrinology.

[12] Hrabovszky E, Liposits Z (2013). Afferent neuronal control of type-I gonadotropin releasing hormone neurons in the human. Front Endocrinol.

[13] Marcondes FK, Bianchi FJ, Tanno AP (2002). Determination of the estrous cycle phases of rats: some helpful considerations. Braz J Biol.

[14] Buchanan TA, Sipos GF, Gadalah S, Yip KP, Marsh DJ, Hsueh W, Bergman RN (1991). Glucose tolerance and insulin action in rats with renovascular hypertension. Hypertension.

[15] Lillie RD (1947). Histopathologic technic and practical histochemistry.

[16] Bhattacharyya S, Khanna S, Chakrabarty K, Mahadevan A, Christopher R, Shankar SK (2009). Anti-brain autoantibodies and altered excitatory neurotransmitters in obsessive–compulsive disorder. Neuropsychopharmacology.

[17] Ghosh NC, Deb C, Banerjee S (1951). Colorimetric determination of epinephrine in blood and adrenal gland. J Biol Chem.

[18] Sangwan RS, Mishra S, Kumar S (1998). Direct Fluorometry of phase-extracted tryptamine-based fast quantitative assay ofl-tryptophan decarboxylase fromCatharanthus roseusLeaf. Anal Biochem.

[19] Basu N, Scheuhammer AM, Rouvinen-Watt K, Evans RD, Trudeau VL, Chan LH (2010). In vitro and whole animal evidence that methylmercury disrupts GABAergic systems in discrete brain regions in captive mink. Comp Biochem Physiol C Toxicol Pharmacol.

[20] MacDonnell P, Greengard O (1975). The distribution of glutamate decarboxylase in rat tissues; isotopic vs fluorimetric assays. J Neurochem.

[21] Yu ZF, Kong LD, Chen Y (2002). Antidepressant activity of aqueous extracts of Curcuma longa in mice. J Ethnopharmacol.

[22] Lee WK, Shin S, Cho SS, Park JS (2000). Purification and characterization of glutamate dehydrogenase as another isoprotein binding to the membrane of rough endoplasmic reticulum. J Cell Biochem.

[23] Ellman P (1956). Pulmonary manifestations in the systemic collagen diseases. Postgrad Med J.

[24] Livak KJ, Schmittgen TD (2001). Analysis of relative gene expression data using real-time quantitative PCR and the 2− ΔΔCT method. Methods.

[25] Maharjan R, Nagar PS, Nampoothiri L (2010). Effect of aloe barbadensis mill. Formulation on Letrozole induced polycystic ovarian syndrome rat model. J Ayurveda Integr Med.

[26] Kauffman AS, Thackray VG, Ryan GE, Tolson KP, Glidewell-Kenney CA, Semaan SJ, Poling MC, Iwata N, Breen KM, Duleba AJ, Stener-Victorin EA (2015). Novel letrozole model recapitulates both the reproductive and metabolic phenotypes of polycystic ovary syndrome in female mice. Biol Reprod.

[27] De Vivo M, Maayani S (1986). Characterization of the 5-hydroxytryptamine1a receptor-mediated inhibition of forskolin-stimulated adenylate cyclase activity in Guinea pig and rat hippocampal membranes. J Pharmacol Exp Ther.

[28] Bhattarai JP, Roa J, Herbison AE, Han SK (2014). Serotonin acts through 5-HT1 and 5-HT2 receptors to exert biphasic actions on GnRH neuron excitability in the mouse. Endocrinology.

[29] He JR, Molnar J, Barraclough CA (1993). Morphine amplifies norepinephrine (NE)-induced LH release but blocks NE-stimulated increases in LHRH mRNA levels: comparison of responses obtained in ovariectomized, estrogen-treated normal and androgen-sterilized rats. Mol Brain Res.

[30] Krieg RJ, Sawyer CH (1976). Effects of intraventricular catecholamines on luteinizing hormone release in ovariectomized-steroid-primed rats. Endocrinology.

[31] Leung PC, Arendash GW, Whitmoyer DI, Gorski RA, Sawyer CH (1982). Differential effects of central adrenoceptor agonists on luteinizing hormone release. Neuroendocrinology.

[32] Jacobi JS, Martin C, Nava G, Jeziorski MC, Clapp C, De La Escalera GM (2007). 17-Beta-estradiol directly regulates the expression of adrenergic receptors and kisspeptin/GPR54 system in GT1-7 GnRH neurons. Neuroendocrinology.

[33] Rubinstein L, Sawyer CH (1970). Role of catecholamines in stimulating the release of pituitary ovulating hormone (s) in rats. Endocrinology.

[34] Plant TM, Zeleznik AJ, editors. Knobil and Neill’s physiology of reproduction. Academic Press; 2014.

[35] Liu X, Herbison AE (2013). Dopamine regulation of gonadotropin-releasing hormone neuron excitability in male and female mice. Endocrinology.

[36] Ciechanowska M, Lapot M, Mateusiak K, Przekop F (2010). Neuroendocrine regulation of GnRH release and expression of GnRH and GnRH receptor genes in the hypothalamus-pituitary unit in different physiological states. Reprod Biol.

[37] Hernández I, Parra A, Méndez I, Cabrera V, del Carmen Cravioto M, Mercado M, Díaz-Sánchez V, Larrea F (2000). Hypothalamic dopaminergic tone and prolactin bioactivity in women with polycystic ovary syndrome. Arch Med Res.

[38] Henderson HL, Townsend J, Tortonese DJ (2008). Direct effects of prolactin and dopamine on the gonadotroph response to GnRH. J Endocrinol.

[39] Kalro BN, Loucks TL, Berga SL (2001). Neuromodulation in polycystic ovary syndrome. Obstet Gynecol Clin N Am.

[40] Gómez R, Ferrero H, Delgado-Rosas F, Gaytan M, Morales C, Zimmermann RC, Simón C, Gaytan F, Pellicer A (2011). Evidences for the existence of a low dopaminergic tone in polycystic ovarian syndrome: implications for OHSS development and treatment. J Clin Endocrinol Metab.

[41] Catalano PN, Di Giorgio N, Bonaventura MM, Bettler B, Libertun C, Lux-Lantos VA (2010). Lack of functional GABA B receptors alters GnRH physiology and sexual dimorphic expression of GnRH and GAD-67 in the brain. Am J Physiol Endocrinol Metab.

[42] Lee K, Porteous R, Campbell RE, Lüscher B, Herbison AE (2010). Knockdown of GABAA receptor signaling in GnRH neurons has minimal effects upon fertility. Endocrinology.

[43] Virmani MA, Stojilković SS, Catt KJ (1990). Stimulation of luteinizing hormone release by gamma-aminobutyric acid (GABA) agonists: mediation by GABAA-type receptors and activation of chloride and voltage-sensitive calcium channels. Endocrinology.

[44] Anderson RA, Mitchell R (1986). Effects of β-aminobutyric acid receptor agonists on the secretion of growth hormone, luteinizing hormone, adrenocorticotrophic hormone and thyroid-stimulating hormone from the rat pituitary gland in vitro. J Endocrinol.

[45] Moore AM, Prescott M, Marshall CJ, Yip SH, Campbell RE (2015). Enhancement of a robust arcuate GABAergic input to gonadotropin-releasing hormone neurons in a model of polycystic ovarian syndrome. Proc Nat Acad Sci USA.

[46] Iremonger KJ, Constantin S, Liu X, Herbison AE (2010). Glutamate regulation of GnRH neuron excitability. Brain Res.

[47] Bourguignon JP, Gerard A, Mathieu J, Simons J, Franchimont P (1989). Pulsatile release of gonadotropin-releasing hormone from hypothalamic explants is restrained by blockade of N-methyl-D, L-aspartate receptors. Endocrinology.

[48] Hrabovszky E, Csapo AK, Kallo I, Wilheim T, Turi GF, Liposits ZS (2006). Localization and osmotic regulation of vesicular glutamate transporter-2 in magnocellular neurons of the rat hypothalamus. Neurochem Int.

[49] Zanisi M, Galbiati M, Messi E, Martini L (1994). The anterior pituitary gland as a possible site of action of kainic acid. Proc Soc Exp Biol Med.

[50] Krsmanovic LZ, Mores N, Navarro CE, Saeed SA, Arora KK, Catt KJ (1998). Muscarinic regulation of intracellular signaling and neurosecretion in gonadotropin-releasing hormone neurons. Endocrinology.

[51] Arai Y, Ishii H, Kobayashi M, Ozawa H (2017). Subunit profiling and functional characteristics of acetylcholine receptors in GT1-7 cells. J Physiol Sci.

[52] Zemkova H, Kucka M, Bjelobaba I, Tomić M, Stojilkovic SS (2013). Multiple cholinergic signaling pathways in pituitary gonadotrophs. Endocrinology.

[53] Ansel L, Bolborea M, Bentsen AH, Klosen P, Mikkelsen JD, Simonneaux V (2010). Differential regulation of kiss1 expression by melatonin and gonadal hormones in male and female Syrian hamsters. J Biol Rhythm.

[54] García-Galiano D, van Ingen Schenau D, Leon S, Krajnc-Franken MA, Manfredi-Lozano M, Romero-Ruiz A, Navarro VM, Gaytan F, van Noort PI, Pinilla L, Blomenröhr M (2012). Kisspeptin signaling is indispensable for neurokinin B, but not glutamate, stimulation of gonadotropin secretion in mice. Endocrinology.

[55] Zhang C, Bosch MA, Rønnekleiv OK, Kelly MJ (2009). γ-aminobutyric acid B receptor mediated inhibition of gonadotropin-releasing hormone neurons is suppressed by kisspeptin-G protein-coupled receptor 54 signaling. Endocrinology.

[56] Goodman RL, Maltby MJ, Millar RP, Hileman SM, Nestor CC, Whited B, Tseng AS, Coolen LM, Lehman MN (2012). Evidence that dopamine acts via kisspeptin to hold GnRH pulse frequency in check in anestrous ewes. Endocrinology.

[57] Neal-Perry G, Lebesgue D, Lederman M, Shu J, Zeevalk GD, Etgen AM (2009). The excitatory peptide kisspeptin restores the luteinizing hormone surge and modulates amino acid neurotransmission in the medial preoptic area of middle-aged rats. Endocrinology.

[58] Drevets WC, Price JL, Furey ML (2008). Brain structural and functional abnormalities in mood disorders: implications for neurocircuitry models of depression. Brain Struct Func.

[59] Darlington CL, Goddard M, Zheng Y, Smith PF (2009). Anxiety-related behavior and biogenic amine pathways in the rat following bilateral vestibular lesions. Ann N Y Acad Sci.

[60] Hasler G (2010). Pathophysiology of depression: do we have any solid evidence of interest to clinicians?. World Psychiatry.

[61] Garcia-Garcia AL, Newman-Taneredi A, Leonardo ED (2013). P5-HT1 a receptors in mood and anxiety: recent insights into autoreceptor versus heteroreceptor function. Psychopharmacology.

[62] Brambilla P, Perez J, Barale F, Schettini G, Soares JC (2003). GABAergic dysfunction in mood disorders. Mol Psychiatry.

[63] Sanacora G, Treccani G, Popoli M (2012). Towards a glutamate hypothesis of depression: an emerging frontier of neuropsychopharmacology for mood disorders. Neuropharmacology.

[64] Kerchner A, Lester W, Stuart SP, Dokras A (2009). Risk of depression and other mental health disorders in women with polycystic ovary syndrome: a longitudinal study. Fertil Steril.

[65] Goodarzi MO, Dumesic DA, Chazenbalk G, Azziz R (2011). Polycystic ovary syndrome: etiology, pathogenesis and diagnosis. Nat Rev Endocrinol.

[66] Aydin M, Yilmaz B, Alcin E, Nedzvetsky VS, Sahin Z, Tuzcu M (2008). Effects of letrozole on hippocampal and cortical catecholaminergic neurotransmitter levels, neural cell adhesion molecule expression and spatial learning and memory in female rats. Neuroscience.

[67] Chaudhari NK, Nampoothiri LP (2017). Neurotransmitter alteration in a testosterone propionate-induced polycystic ovarian syndrome rat model. Horm Mol Biol Clin Investig.

